# Understanding the performance and reliability of NLP tools: a comparison of four NLP tools predicting stroke phenotypes in radiology reports

**DOI:** 10.3389/fdgth.2023.1184919

**Published:** 2023-09-28

**Authors:** Arlene Casey, Emma Davidson, Claire Grover, Richard Tobin, Andreas Grivas, Huayu Zhang, Patrick Schrempf, Alison Q. O’Neil, Liam Lee, Michael Walsh, Freya Pellie, Karen Ferguson, Vera Cvoro, Honghan Wu, Heather Whalley, Grant Mair, William Whiteley, Beatrice Alex

**Affiliations:** ^1^ Advanced Care Research Centre, Usher Institute, University of Edinburgh, Edinburgh, United Kingdom; ^2^ Centre for Clinical Brain Sciences, University of Edinburgh, Edinburgh, United Kingdom; ^3^ School of Informatics, University of Edinburgh, Edinburgh, United Kingdom; ^4^ Canon Medical Research Europe Ltd., AI Research, Edinburgh, United Kingdom; ^5^ School of Computer Science, University of St Andrews, St Andrews, United Kingdom; ^6^ School of Engineering, University of Edinburgh, Edinburgh, United Kingdom; ^7^ Medical School, University of Edinburgh, Edinburgh, United Kingdom; ^8^ Intensive Care Department, University Hospitals Bristol and Weston, Bristol, United Kingdom; ^9^ National Horizons Centre, Teesside University, Darlington, United Kingdom; ^10^ School of Health and Life Sciences, Teesside University, Middlesbrough, United Kingdom; ^11^ Department of Geriatric Medicine, NHS Fife, Fife, United Kingdom; ^12^ Institute of Health Informatics, University College London, London, United Kingdom; ^13^ Alan Turing Institute, London, United Kingdom; ^14^ Generation Scotland, Institute of Genetics and Cancer, University of Edinburgh, Edinburgh, United Kingdom; ^15^ Neuroradiology, Department of Clinical Neurosciences, NHS Lothian, Edinburgh, United Kingdom; ^16^ Edinburgh Futures Institute, University of Edinburgh, Edinburgh, United Kingdom; ^17^ School of Literatures, Languages and Cultures, University of Edinburgh, Edinburgh, United Kingdom

**Keywords:** natural language processing, brain radiology, stroke phenotype, electronic health records

## Abstract

**Background:**

Natural language processing (NLP) has the potential to automate the reading of radiology reports, but there is a need to demonstrate that NLP methods are adaptable and reliable for use in real-world clinical applications.

**Methods:**

We tested the F1 score, precision, and recall to compare NLP tools on a cohort from a study on delirium using images and radiology reports from NHS Fife and a population-based cohort (Generation Scotland) that spans multiple National Health Service health boards. We compared four off-the-shelf rule-based and neural NLP tools (namely, EdIE-R, ALARM+, ESPRESSO, and Sem-EHR) and reported on their performance for three cerebrovascular phenotypes, namely, ischaemic stroke, small vessel disease (SVD), and atrophy. Clinical experts from the EdIE-R team defined phenotypes using labelling techniques developed in the development of EdIE-R, in conjunction with an expert researcher who read underlying images.

**Results:**

EdIE-R obtained the highest F1 score in both cohorts for ischaemic stroke, ≥93%, followed by ALARM+, ≥87%. The F1 score of ESPRESSO was ≥74%, whilst that of Sem-EHR is ≥66%, although ESPRESSO had the highest precision in both cohorts, 90% and 98%. For F1 scores for SVD, EdIE-R scored ≥98% and ALARM+ ≥90%. ESPRESSO scored lowest with ≥77% and Sem-EHR ≥81%. In NHS Fife, F1 scores for atrophy by EdIE-R and ALARM+ were 99%, dropping in Generation Scotland to 96% for EdIE-R and 91% for ALARM+. Sem-EHR performed lowest for atrophy at 89% in NHS Fife and 73% in Generation Scotland. When comparing NLP tool output with brain image reads using F1 scores, ALARM+ scored 80%, outperforming EdIE-R at 66% in ischaemic stroke. For SVD, EdIE-R performed best, scoring 84%, with Sem-EHR 82%. For atrophy, EdIE-R and both ALARM+ versions were comparable at 80%.

**Conclusions:**

The four NLP tools show varying F1 (and precision/recall) scores across all three phenotypes, although more apparent for ischaemic stroke. If NLP tools are to be used in clinical settings, this cannot be performed “out of the box.” It is essential to understand the context of their development to assess whether they are suitable for the task at hand or whether further training, re-training, or modification is required to adapt tools to the target task.

## 1. Introduction

Natural language processing (NLP) can support the automated reading of radiology reports (1, 2). Research on clinical NLP has focused on improving the phenotyping of coded health data routinely collected during healthcare visits (3), enhancing cohort identification for research studies (4, 5), and improving healthcare quality (6, 7). However, a gap in translation from research to clinical application remains.

Deploying NLP tools out of the box in clinical settings is challenging because these tools are often built on datasets with limited population characteristics. This issue emerges because access to large, diverse datasets representative of demographics and clinical contexts is restricted because of the highly sensitive nature of these data and privacy concerns in releasing data for research. Limited access to data also inhibits the validation of NLP tools on datasets beyond those used to train and develop them. There are some accessible free-text radiology electronic health records (EHRs) from the US that researchers can use, but these are different from UK healthcare data and may result in NLP tools that do not work well in the UK context (8). In addition, initiatives such as those of Mitchell et al. (9) and Bender and Friedman (10) have seen limited uptake within the clinical NLP community. These initiatives support more transparent and uniform methods of sharing information about the intended use of a model and minimising its use in contexts where it would not be appropriate. Furthermore, employing these frameworks can address issues related to exclusion and bias, resulting in models with improved generalisation abilities. Reluctance to embrace these frameworks could be because of the lack of access to the linked demographic data required. Nonetheless, it could be a useful approach to help support the transition of NLP tools to clinical use.

Our study, which compares four NLP tools across two different cohorts, highlights how hard it is to deploy NLP tools out of the box. We show the importance of gaining a deeper understanding of the context a model is developed within and how this context relates to its performance and usage parameters. The two cohorts we used are (1) a study of patients with delirium in one UK hospital system (NHS Fife) and (2) a population-based cohort study (Generation Scotland), spanning multiple National Health Service (NHS) Scotland health boards (11). The four off-the-shelf NLP tools compared (EdIE-R, ALARM+, ESPRESSO, and Sem-EHR) include rule-based and neural methods and focused on three phenotypes found in brain radiology reports, namely, ischaemic stroke, small vessel disease (SVD), and atrophy. We include two versions of ALARM+, with one including uncertainty predictions. We evaluate the performance of these tools in comparison to clinical experts annotating the original radiology reports and research radiologists reading the brain images. Our findings reveal the differences in demographic variables, such as age and language used in reporting. In addition, the geographical areas of reports and the clinical settings under which the annotation labels (ground truth) are applied all influence how NLP tools perform when faced with new data.

## 2. Material and methods

### 2.1. Data cohorts

We utilised two data cohorts, and all data were obtained from the Scottish NHS health boards^1^. NHS health boards are responsible for delivering frontline healthcare services within specific regions of Scotland. Clinically, there will be regional differences in the data, for example, between urban and rural areas and richer and poorer areas. There may also be linguistic differences and variations in data recording styles because of local conventions or individual writing styles.

*NHS Fife* is a cohort of acute medical inpatients aged >65 years admitted to a district general hospital who underwent an Older People Routine Acute Assessment and a head CT scan reported by a radiologist as part of their clinical care (routine clinical report). Following data linkage, this dataset contained 2,345 routine clinical reports with corresponding research image reads. Age was collected during the time of the scan. Following data pre-processing of the routine clinical reports (described in Section 2.3), we selected 750 CT reports for annotation in our study, with the selection of this number constrained by limits on annotation time.

*Generation Scotland*^2^ *(GS)* is a cohort of 24,000 participants from both the Scottish Family Health Study and the 21st Century Genetic Health study. This cohort consists of data from different Scottish NHS health boards. The data from NHS Fife are combined with GS data from NHS Tayside—we denote this subset as *Tayside & Fife* data throughout the paper. We received routine clinical brain imaging reports for all brain scans conducted on GS participants since these reports began to be stored digitally (around the year 2007 for most boards to 2021). After data pre-processing (described in Section 2.3), the final number of CT reports used in this study was 1,487 (635 from Greater Glasgow and Clyde, 368 from Grampian, 341 from Tayside & Fife, and 70 from Lothian). Age was collected during the time of the scan.

### 2.2. Data annotation

**Figure 1:**
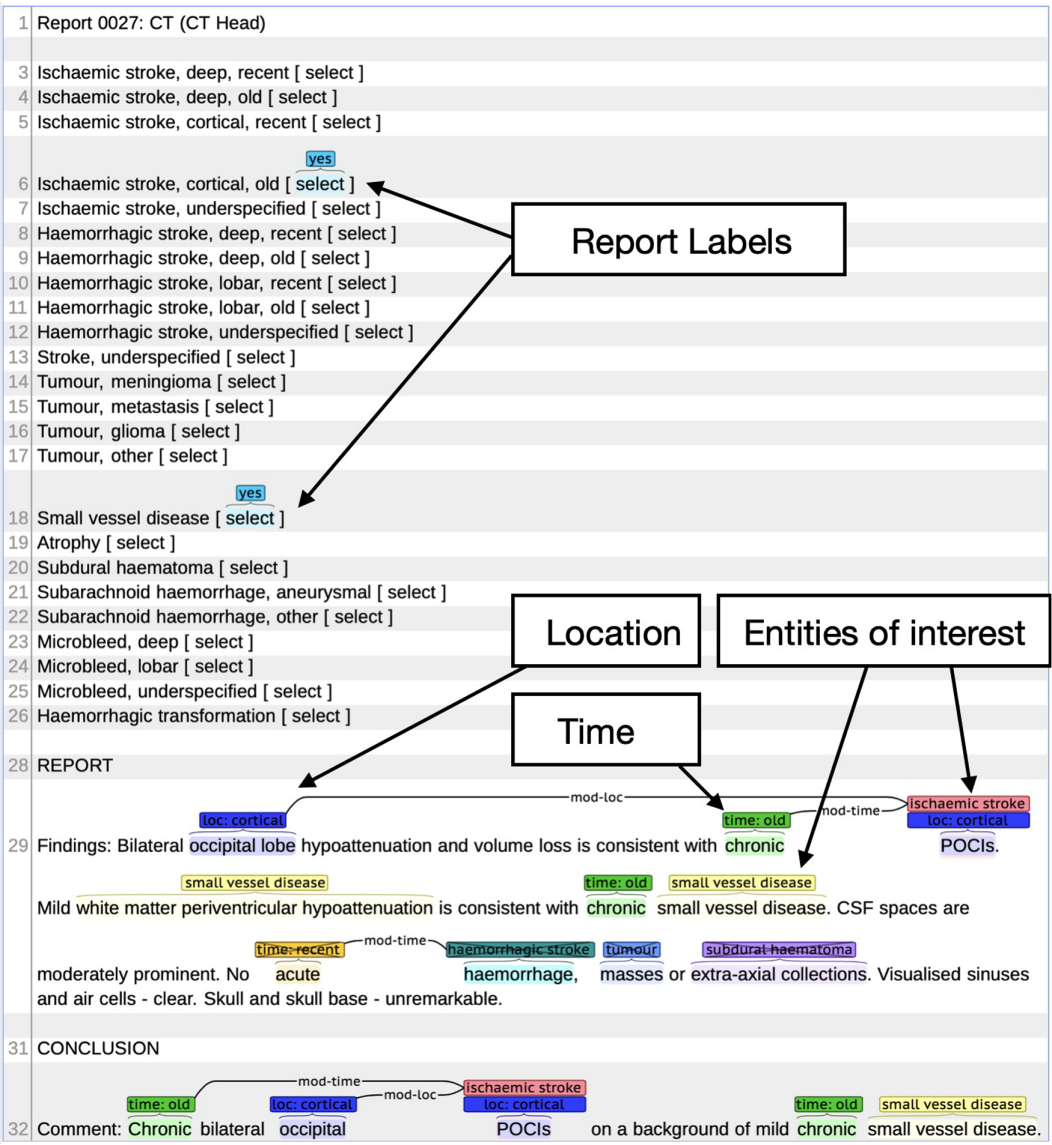
Example radiology report in BRAT annotation tool. Example shows the document (report labels) and individual phenotype labels (location, entities of interest, and time).

Alex et al. (12) reported the annotation schema used in this study. We defined three phenotypes, namely, any ischaemic stroke (cortical or deep, old or recent, or unspecified ischaemic stroke), atrophy, and SVD. Using this labelling schema with the BRAT annotation tool (13), annotators highlighted named entities within the text and any related modifiers of location and time and annotated negation, if present. The annotator then selected the relevant document-level phenotype labels (see an annotation example in Figure 1).

Training was carried out for all annotators on 115 files from the subset of GS for NHS Tayside & Fife in an iterative process involving weekly meetings to discuss discrepancies and answer questions. The annotators consist of a consultant neurologist, a clinical fellow, a junior doctor, a clinical laboratory scientist, and a medical student. The consultant neurologist and clinical fellow performed the NHS Fife annotation, with a 10% overlap for double annotation. All annotators conducted the GS annotation on each NHS health board within the GS dataset, with a 10% overlap on all NHS health boards, except for NHS Lothian, which was double annotated. Supplementary 1 Tables S1, S2 report the kappa values for annotators for atrophy, SVD, and subtype level for ischaemic stroke. The consultant neurologist resolved any disagreements in the double annotated data.

### 2.3. Data pre-processing

**Figure 2:**
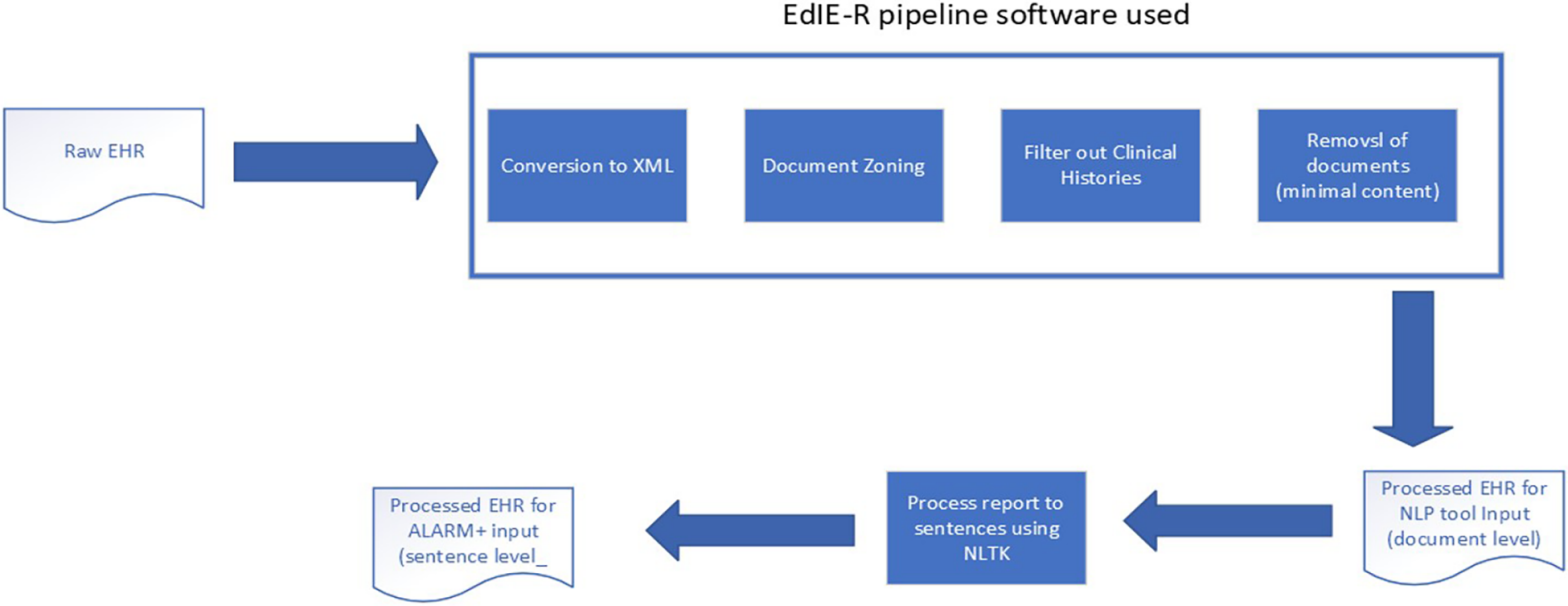
Data processing pipeline.

The raw reports and associated meta-fields from each study, supplied in CSV, were prepared with parts of the EdIE-R pipeline, as Alex et al. (12) described, by converting text into XML and identifying section structures such as clinical history, report, and conclusion (see Figure 2). During the process, if a report was found to contain no or minimal text, e.g., “see previous report,” it was excluded from this study. The clinical history sections were filtered out. Therefore, only the body of a report and the conclusion were used as input into any NLP tool in predicting outcomes. The ALARM+ input underwent further processing using the Python NLTK^3^ package to split the document-level report into sentences.

### 2.4. Research image reads

An expert researcher for the NHS Fife cohort read the brain images. They recorded infarcts, SVD, and atrophy in a standardised way as part of a study to understand neuroimaging correlates of delirium (we refer to this as a research image read). Two expert neurologists mapped these research image reads to the three stroke phenotypes. Supplementary 2 shows the values of the research image reads recorded and how these were mapped to the three stroke phenotypes.

### 2.5. NLP tools

Four different tools that predict cerebrovascular phenotypes were used, namely, EdIE-R, Sem-EHR, ESPRESSO, and ALARM+. All tools were used out of the box and not modified for this study. For the ALARM+ model, we compare two output configurations.

EdIE-R^4^ is a rule-based system that first performs named entity recognition, negation detection, and relation extraction and then classifies reports at a document level using 13 phenotypes and two time and two location modifier labels. The rules for using this tool were originally developed on radiology reports of a consented cohort of the Edinburgh Stroke Study for NHS Lothian patients using both CT and MRI scans and further fine-tuned on routine radiology reports from NHS Tayside (12, 14). Whilst data from these NHS health boards are included in GS, the likelihood of an overlap for NHS Lothian is very low because the GS cohort includes patients of all ages and not specifically those who suffered from stroke. In addition, the data for the stroke study were collected before 2008, and most of its patients have already passed away. It is possible for NHS Tayside data to be included in the GS data. However, we cannot verify duplicates because these datasets were processed in separate safe research environments as part of different projects and cannot be merged because of data governance. This study uses the annotation schema developed in conjunction with this tool.

Sem-EHR^5^ identifies various biomedical concepts within the clinical text based on the Unified Medical Language system. It then uses rules to determine if the phenotypes of interest are present at a document level. The version used in this work was developed mainly on CT scans with a small number of MRI scans using data from EMR in UK NHS Trusts in London and further extended on Scottish imaging datasets. Rannikmäe et al. (3) described the model.

ESPRESSO is a rule-based system designed to identify silent brain infarction (SBI) and white matter disease from radiology reports. The tool was developed from multi-site hospital data within the US using both CT and MRI scans (15, 16).

ALARM+ (17) is a per-label attention neural network model based on PubMedBERT. This model was developed on an NHS Glasgow and Greater Clyde dataset from patients diagnosed with stroke, as extracted for the Industrial Centre for AI Research in Digital Diagnostics project.^6^ This part of the project aimed to detect indications and contra-indications for giving thrombolysis treatment to patients with acute stroke. ALARM+ was trained on anonymised radiology reports corresponding to non-contrast head CT scans from the stroke event and the 18 months preceding and following that event and on synthetic text data (18). This model provides sentence-level predictions regarding whether a phenotype is *present, negative, uncertain,* or *not mentioned*. In two ways, we evaluate two output configurations for the ALARM+ model by aggregating the sentence-level outputs to a document-level output in two ways. The first is referred to as ALARM+, without uncertainty, where we only take the predictions of a phenotype being *present.* The second is ALARM+U, with uncertainty, where we consider both the *present* and *uncertain* predictions to indicate that the phenotype is present.

### 2.6. Mapping NLP tool outcomes to phenotypes

One of the challenges of this work was that the NLP tools have all been developed according to varying annotation schemas. For this work, we focused on the common predictions between them, including ischaemic stroke (all types), SVD, and atrophy. Clinically, our phenotypes of interest are related. They all occur more frequently with advancing age and have similar lifestyle and genetic risk factors, but they are readily distinguishable on imaging. Patients may develop one of these conditions or any combination. EdIE-R, ALARM+, and Sem-EHR identify all three outcomes. In contrast, ESPRESSO only predicts SBI, which is an asymptomatic ischaemic stroke seen on imaging (CT or MRI scan). Thus, the clinical picture is relevant in determining SBI. In the ESPRESSO study, the cohort consisted of patients who did not have any clinically evident stroke any time before or up to 30 days after the imaging exam (15).

Except ALARM+, all tools generate document-level output to determine whether a phenotype was present in each report. Sentence-level predictions for ALARM+ were aggregated to establish document-level labels. In cases where there were contradictory sentence-level predictions about the presence or negation of a phenotype in ALARM+ output, we marked the phenotype as present. This approach also matched the supervision (labelling) performed by ALARM+ authors where word-level contradictions occurred in a sentence.

### 2.7. Comparisons and performance measures

Comparisons of performance by phenotype across GS and NHS Fife cohorts were carried out for each NLP tool and the three phenotypes. We reported the F1 score (harmonic mean of precision and recall), precision (also known as positive predictive value, i.e., PPV), and recall. Metrics were reported for the total populations and subdivided within the GS dataset based on age groups (<50, 50–70, and 71+ years) and the NHS health board. We did not divide the NHS Fife data by age group since all the patients were >65 years of age, and only 4% (or 35 records) from the NHS Fife data were below 71 years. The Supplementary Material presents the 95% confidence intervals (CIs) for results. We used CIs estimated using a beta distribution and calculated them using the Python package scipy.stats^7^ and the beta.ppf function across the reports to give readers an approximation of CIs. Our decision to apply this method is based on the work by Goutte and Gaussier (19), which compares the results of different methods for precision and recall. They argue that summary statistics such as precision and recall do not always correspond to sample medians or means. Thus, a bootstrap method may fail to give accurate CIs. Their approach instead estimates the distribution for precision and recall and uses this to compare results for different methods. Precision∼*Be* (*TP* + *λ*, *FP* + *λ*) Recall∼*Beta* (*TP* + *λ*, *FN* + *λ*), where *λ* is an adjustment factor for prior information, and we assume uniform priors (1,1) for both precision and recall. However, there is no consensus in the field yet regarding the derivation of CIs. Supplementary 3 provides the CIs for the breakdown by age and NHS health board. However, this breakdown leads to small partitions of data with even smaller numbers of people having one or more of the three phenotypes, leading to broad CIs, which can make it difficult to draw definitive conclusions based on the reported CIs. Nonetheless, we provide them for completeness.

Using research image reads from NHS Fife, we compare each NLP tool to the research image read by phenotype and compare the human-annotated labels to the research image reads using the research image reads as ground truth. We also compare the NLP tools to the human annotations using the annotations as ground truth.

## 3. Results

### 3.1. Prevalence of phenotypes by annotated labels

Table 1 shows the prevalence rate of the three phenotypes, using the annotated labels, and the distribution of scans by age group for both cohorts. More SVD and atrophy than ischaemic stroke are identified in both cohorts, particularly in NHS Fife. This finding is expected as NHS Fife is a cohort for elderly patients with delirium; 95% of the scans were from patients aged 71 or above, whereas only 37% of the patients in the FS data were in this age range.

**Table 1:** Prevalence rate of all phenotypes in NHS Fife and Generation Scotland cohorts, numbers of scans with phenotype present, % of total number of scans with phenotype present out of the full number of scans in the dataset, and number of scans in each age group per data cohort.

	NHS Fife	Generation Scotland
Phenotype		
Ischaemic stroke (all)	272 (37.50%)	225 (15.13%)
Small vessel disease	488 (67.40%)	362 (24.34%)
Atrophy	476 (65.75%)	308 (20.71%)
Age group (years)		
<50	0	293
50–70	35	650
71+	686	544

### 3.2. Ischaemic stroke

#### 3.2.1. F1 performance

**Figure 3:**
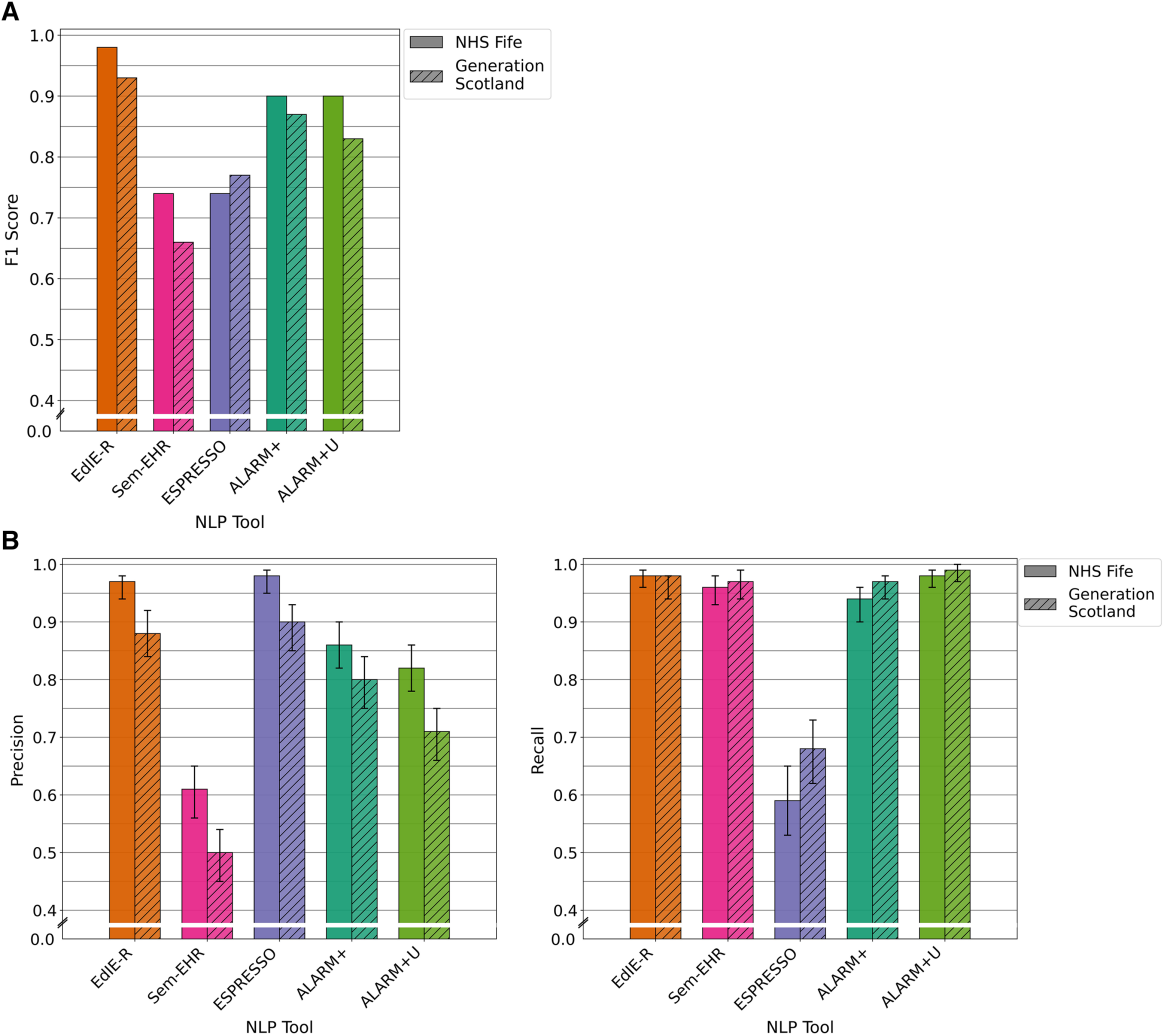
(**A**) F1 performance of NLP tools on both NHS Fife and Generation Scotland data for ischaemic stroke. (**B**) Precision and recall performance of NLP tools on both NHS Fife and Generation Scotland data for ischaemic stroke and 95% CIs.

The four tools had varying F1 scores, and all performance levels dropped in the GS data compared with that of NHS Fife, except ESPRESSO. EdIE-R had the highest F1 score on both cohorts at ≥93%, with Sem-EHR scoring the lowest at ≥66%. ESPRESSO achieved the same performance level as Sem-EHR on the NHS Fife cohort, both scoring 74%, which increased to 77% on the GS data. Both versions of ALARM+ performed the same on the NHS Fife, with scores of 90%, but differed on the GS, where ALARM+ scored 87% and ALARM+U scored 83% (Figure 3A).

#### 3.2.2. Precision and recall performance

In both cohorts, EdIE-R had a fairly high precision of ≥88% and high recall of ≥98%. Sem-EHR had a low precision of ≥50% but a high recall of ≥96%. ESPRESSO had the highest precision of all tools in both cohorts at ≥90% but the lowest recall in both cohorts at ≥58%. ALARM+ and ALARM+U had a modest precision of 80% and 71%, respectively, and high recall at ≥94% (Figure 3B).

#### 3.2.3. Performance across age groups

All the tools had lower F1 scores in the *<50* age group except ALARM+, which performed best in this group with a score of 92%. However, when uncertainty was added to ALARM+U, the performance dropped to 12% compared with that without uncertainty. EdIE-R had the highest F1 score in the *50–70* and *71*+ age groups at 92% and 91%, respectively, closely followed by ALARM+, which was 5% lower in the 50–70 group and only 1% lower in the *71*+ age group. Both Sem-EHR and ESPRESSO performed better as the age group increased, with Sem-EHR rising 13%, from 57% to 70%, and ESPRESSO 18%, from 63% to 81%, respectively, across the age groups (Table 2).

**Table 2:** NLP tool F1 score (%) for ischaemic stroke performance across age groups (<50, 50–70, and 71+ years) and NHS health boards (Tayside & Fife, Lothian, GGC, and Grampian) in Generation Scotland data, excluding the NHS Fife dataset.

Age group	EdIE-R	Sem-EHR	ESPRESSO	ALARM+	ALARM+U
<50	0.89	0.57	0.63	**0** **.** **92**	0.80
50–70	**0** **.** **92**	0.61	0.73	0.87	0.81
71+	**0** **.** **91**	0.70	0.81	0.90	0.84
Tayside & Fife	**1** **.** **0**	0.77	0.76	0.86	0.85
Lothian	**1** **.** **0**	0.42	0.73	0.87	0.81
GGC	0.89	0.67	0.75	0.88	**0** **.** **92**
Grampian	**0** **.** **91**	0.63	0.82	0.87	0.83

The precision, recall, and 95% CIs can be found in Supplementary 3. Bold figures indicate the highest F1 score.

#### 3.2.4. Performance across health boards

EdIE-R performed best in the NHS health boards it was trained and tuned on (Lothian and Tayside & Fife), with a 100% F1 score, which dropped to 9% in NHS Grampian and 11% in GCC. ALARM+ performed more consistently than EdIE-R across the health boards, varying 2%, from 86% to 88%. ALARM+U varied more, with the lowest score in Lothian at 81% and highest score in GCC (the health board it was trained on) at 92%. The performance of Sem-EHR and ESPRESSO varied across the health boards, both lowest on Lothian at 42% and 73%, respectively. Sem-EHR performed higher on Tayside & Fife with 77% compared with 76%, and ESPRESSO performed 8% higher than Sem-EHR on GCC at 75% and 19% higher on Grampian (Table 2).

#### 3.2.5. Error analysis

All tools had more false positives in the GS data compared with NHS Fife, largely due to words or phrases that do not clearly indicate whether a phenotype was present and are open to annotator interpretation, e.g., “This *may be* an old infarct.” These phrases were found more often in scans of younger people. Therefore, this finding reflected the differences in age between the two cohorts. For most of these false positives, we found that the annotator would mark the phenotype not present, whereas the tool would take it as a positive incidence. EdIE-R was less likely to do this as it was designed with the annotation guidelines used in this study. Emphasising this problem of uncertainty, adding uncertainty with ALARM+U increases false positives even further.

ESPRESSO recall was expected to score lower as this tool only predicts asymptomatic brain infarctions, which means it is more likely to miss more instances, resulting in higher false negatives.

Another factor in the lower precision for ALARM+ in predicting ischaemic stroke was the difference in the definitions of the labelling model. ALARM+ identified phrases as ischaemic stroke that annotators labelled as small vessel diseases, e.g., “*ischaemic change*” and “*micro-vascular change.*”

### 3.3. SVD

#### 3.3.1. F1 performance

**Figure 4:**
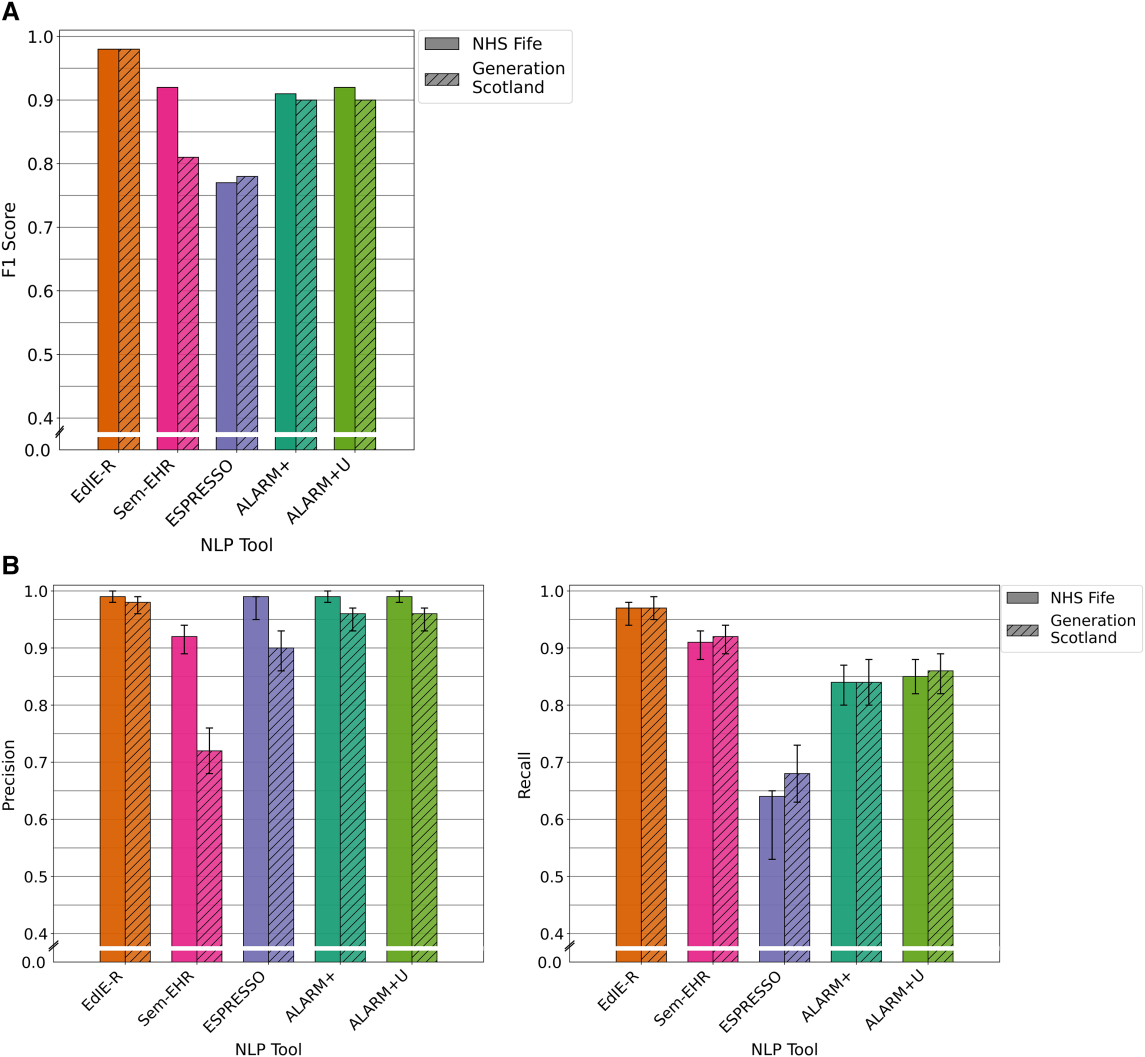
(**A**) F1 performance of NLP tools on both NHS Fife and Generation Scotland data for SVD. (**B**) Precision and recall performance of NLP tools on both NHS Fife and Generation Scotland data for SVD and 95% CIs.

For the prediction of SVD on reports of brain CT scans, there was very little difference in the performance between the two cohorts for each tool except for Sem-EHR. EdIE-R had the highest F1 score of 98% in both cohorts. Sem-EHR scored high at 92% but dropped to 81% on GS. ESPRESSO was lowest in both cohorts at 77%, rising 1% on GS. ALARM+ had a high F1 score of ≥90% over both cohorts, with very little difference, 1%, between the two ALARM+ versions (Figure 4A).

#### 3.3.2. Precision and recall performance

EdIE-R had a high precision of ≥98% and a recall of ≥97%. Sem-EHR had a modest to higher precision, 72% and 92%, but a high recall of ≥91%. ESPRESSO has a high precision of ≥90% but a low recall of 68%, and ALARM+ and ALARM+U had a high precision of ≥96% and modest recall of ≥85% (Figure 4B).

#### 3.3.3. Performance across age groups

EdIE-R performed best across age groups with the highest F1 scores, although it is 9% lower in the *<50* group compared with the *71+ group*. The difference in F1 performance was much higher for all the other tools when comparing the *<50* group to the *71+* group. There was an 80% difference across the age groups in F1 score for Sem-EHR from 8% to 88%, 51% for ESPRESSO from 29% to 80%, 52% for ALARM+ from 40% to 92%, and 53% for ALARM+U from 40% to 93% (Table 3).

**Table 3:** NLP tool F1 score (%) for SVD performance across age groups (<50, 50–70, and 71+ years) and NHS health boards (Tayside & Fife, Lothian, GGC, and Grampian) in Generation Scotland data, excluding NHS Fife dataset.

Age group	EdIE-R	Sem-EHR	ESPRESSO	ALARM+	ALARM+U
<50	**0** **.** **89**	0.08	0.29	0.40	0.40
50–70	**0** **.** **96**	0.72	0.73	0.85	0.86
71+	**0** **.** **98**	0.88	0.80	0.92	0.93
Tayside & Fife	**0** **.** **99**	0.73	0.68	0.83	0.83
Lothian	**1** **.** **0**	0.49	0.88	0.88	0.91
GGC	**0** **.** **97**	0.88	0.67	0.91	0.92
Grampian	**0** **.** **98**	0.80	0.89	0.89	0.90

The precision, recall, and 95% CIs can be found in Supplementary 3. Bold figures indicate the highest F1 score.

#### 3.3.4. Performance across health boards

EdIE-R performs the best across all health boards at ≥98%. Sem-EHR performance was lowest on Lothian data at 49%, rising to 88% in GCC. ESPRESSO performed the same as ALARM+ on Lothian, 88% and 89% on Grampian, but lower on Tayside & Fife at 68% and 67% on GCC. ALARM+U outperformed ALARM+ by a small margin on every health board but Tayside & Fife, with both versions reaching ≥83% (Table 3).

#### 3.3.5. Error analysis

SVD is often mentioned in relation to age (e.g., “*normal for age*”), and this led to some tools predicting SVD even when the annotator did not label it as such because it was not considered pathological. EdIE-R was again at an advantage, tuned to the annotation schema used. Sem-EHR also incorrectly labelled mentions of the word “vessel” and related abbreviations as SVD, which increased false positives.

The lower recall for ALARM+ is again an artefact of the annotation schema used and shows the opposite effect of that seen in predicting ischaemic stroke. Texts that an annotator would label as SVD are labelled by ALARM+ as ischaemic stroke, creating more false negative predictions.

### 3.4. Atrophy

#### 3.4.1. F1 performance

**Figure 5:**
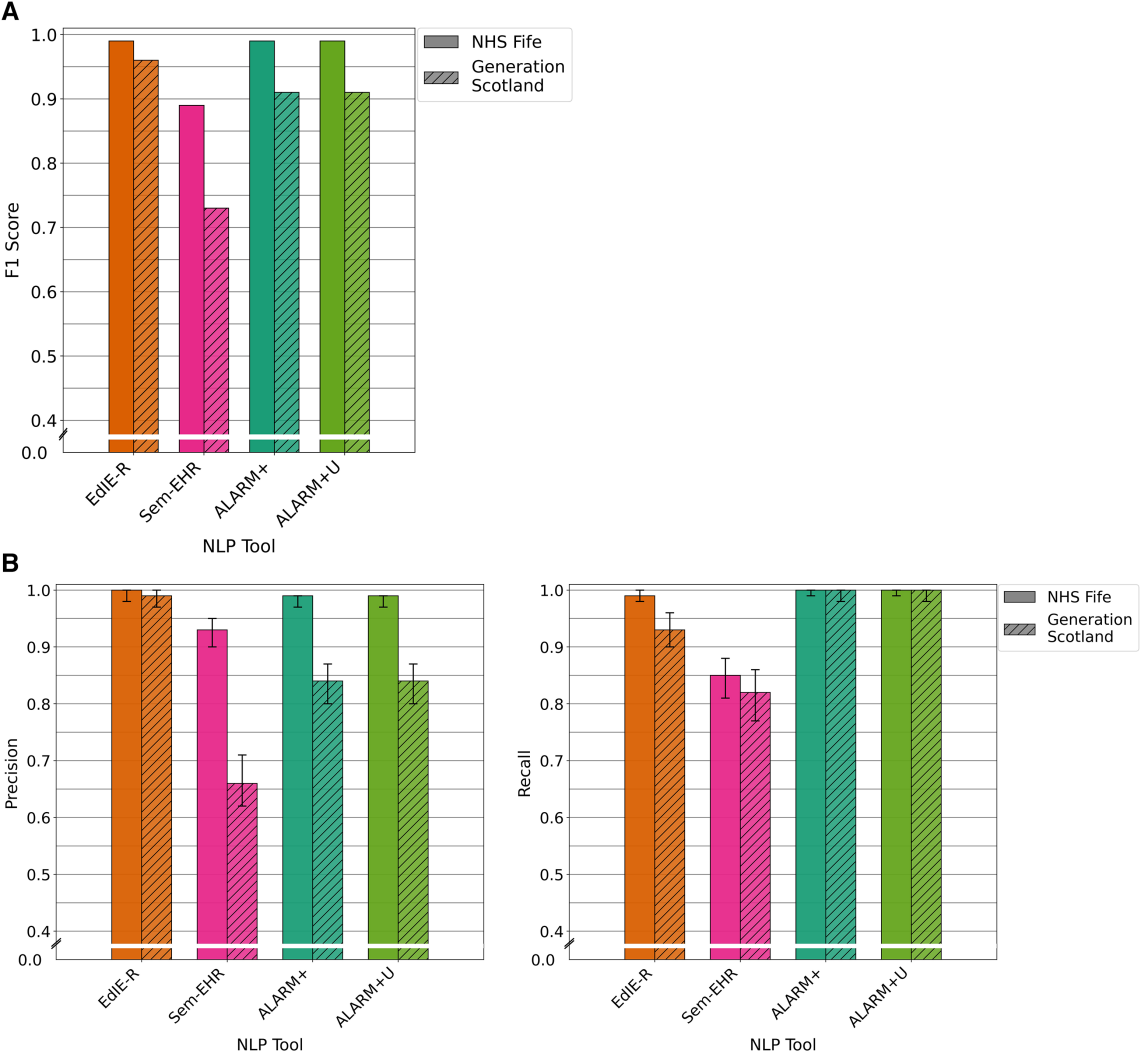
(**A**) F1 performance of NLP tools on both NHS Fife and Generation Scotland data for atrophy. (**B**) Precision and recall performance of NLP tools on both NHS Fife and Generation Scotland data for atrophy and 95% CIs.

For the prediction of atrophy on reports of brain CT scans, EdIE-R and both versions of ALARM+ achieved identical scores of 99% on NHS Fife. However, their performance declined in GS, with EdIE-R dropping to 96% and both ALARM+ versions dropping to 91%. Sem-EHR scored lowest at 89% on NHS Fife and 73% on GS (Figure 5A).

#### 3.4.2. Precision and recall performance

EdIE-R had high precision of ≥98% and recall of ≥93%. Sem-EHR had a modest precision, 72% and 92% but a high recall of ≥91%. ALARM+ and ALARM+U had high precision in NHS Fife at 99%, dropping to 84% in GS, but have the highest recall in both datasets at 100% (Figure 5B).

#### 3.4.3. Performance across age groups

EdIE-R performed more consistently across the age groups, differing by 6%, starting at 94% in the <50 group, rising to 100% in the *50–70 group*, and dropping again to 96% in the *71+* group. Sem-EHR performed much lower than both tools at 26% in the <50 group, rising to 80% in the *71+* age group*.* Both versions of ALARM+ scored the same but varied 21% across the age groups, starting at 72% (*<50* age group) and rising to 93% for the *71+* age group (Table 4).

**Table 4:** NLP tool F1 score (%) for atrophy performance across age groups (<50, 50–70, and 71+ years) and NHS health boards (Tayside & Fife, Lothian, GGC, and Grampian) in Generation Scotland data, excluding the NHS Fife dataset.

Age group	EdIE-R	Sem-EHR	ALARM+	ALARM+U
<50	**0** **.** **94**	0.26	0.72	0.72
50–70	**1** **.** **0**	0.68	0.87	0.87
71+	**0** **.** **96**	0.80	0.93	0.93
Tayside & Fife	**0** **.** **98**	0.79	0.96	0.96
Lothian	**1** **.** **0**	0.29	0.81	0.81
GGC	**0** **.** **95**	0.70	0.93	0.93
Grampian	**0** **.** **97**	0.83	0.87	0.87

The precision, recall, and 95% CIs can be found in Supplementary 3. Bold figures indicate the highest F1 score.

#### 3.4.4. Performance across health boards

Again, EdIE-R performed best in the health boards it was trained and tuned on, scoring 100% in Lothian and dropping to 95% in GCC. Sem-EHR, as with all the other phenotypes, performed lowest on the Lothian data at 29% and much higher on the other health boards, reaching 83% in Grampian. Both ALARM+ versions performed the same across all the health boards, lowest on Lothian 81% and reaching 96% in Tayside & Fife (Table 4).

#### 3.4.5. Error analysis

Similar to SVD, atrophy is often mentioned in relation to the age of a patient. Sem-EHR and ALARM+ correctly label statements such as “*mild age related generalised cerebral atrophy*” as indicating atrophy, but they also label “*brain volume is preserved for age*” and “*cerebral volume is normal for age*” as indicating the presence of atrophy. In contrast, the annotator can distinguish that these phrases state that atrophy is not present. Both instances occur more in the GS data and younger patients and are the main reason for more false positives, resulting in worse precision scores between the two cohorts and in age groups.

### 3.5. Research image reads

#### 3.5.1. Data description of research image reads

More instances of ischaemic stroke are identified in the brain image reads than what human annotators have identified in the reports (Table 5). Approximately 70% of these additional ischaemic stroke labels are “*old ischaemic strokes.*” Routine clinical reports do not always contain every aspect of what might be included in a brain image read. For example, a radiologist reading a routine clinical image may have prior information about old strokes from existing images or, conversely, address a specific request and leave out details not pertinent to the request at hand. This largely accounts for the discrepancies between the image reads and annotated labels.

**Table 5:** The number of phenotypes labelled during the research image reads of the brain image and the number of phenotypes labelled during human annotation of the radiology reports and precision and recall values for each phenotype comparing research image read as ground truth to human-annotated labels in reports.

Phenotype	Image	Report	Precision	Recall
Ischaemic stroke	459	272	0.88	0.53
Small vessel disease	548	488	0.90	0.80
Atrophy	516	476	0.83	0.77

#### 3.5.2. Comparison of results for research image reads

Table 5 shows the precision and recall when comparing research image reads as ground truth to human-annotated labels in the radiology reports. In contrast to the results of the NLP tools and human-annotated labels, atrophy precision is lowest at 83% and SVD is highest at 90%. The recall for SVD and atrophy was higher than that for ischaemic stroke, which is expected as we know more *old ischaemic strokes* were found in the brain image reads than those mentioned in the reports.

Table 6 compares research image reads to NLP tool outputs. Compared with the results of NLP tools against the human-annotated labels, all tools are much more comparable, with almost no difference between EdIE-R and both ALARM+ versions in atrophy and Sem-EHR being much closer in performance. Recall was high for ALARM+ but declined by 18% in ALARM+U when we added uncertainty for ischaemic stroke. Notably, the performance of ALARM+ measured from F1 was much higher than that of EdIE-R because of the better recall. Sem-EHR and EdIE-R were much closer in performance compared with human report labels in SVD, with ALARM+ having the highest precision.

**Table 6:** The precision, recall, and F1 scores comparing the NLP tools for each phenotype to the brain image reads.

	Ischaemic stroke			Small vessel disease			Atrophy		
	Precision	Recall	F1	Precision	Recall	F1	Precision	Recall	F1
EdIE-R	0.88	0.53	0.66	0.90	0.78	**0** **.** **84**	0.83	0.77	**0** **.** **80**
Sem-EHR	0.77	0.72	**0** **.** **74**	0.87	0.77	0.82	0.81	0.68	0.74
ESPRESSO	0.89	0.32	0.47	0.92	0.53	0.67	–	–	–
ALARM+	0.85	0.57	0.68	0.91	0.69	0.78	0.83	0.78	**0** **.** **80**
ALARM+U	0.84	0.59	0.69	0.91	0.70	0.79	0.83	0.78	**0** **.** **80**

Bold figures indicate the highest F1 score.

Comparing Tables 5 and 6 for ischaemic stroke, the EdIE-R precision performance is identical to that of human annotators, and ESPRESSO precision is 1% higher. Comparing recall in this way, Sem-EHR and both versions of ALARM+ exceed the recall of human annotators. For SVD, EdIE-R, ESPRESSO, and both ALARM+ versions have the same or slightly higher precision than human annotators, but the recall of all NLP tools is lower than that of human annotators. Finally, for atrophy, EdIE-R matches the precision and recall of human annotators, and both ALARM+ versions match the performance of human annotators in precision and perform better in recall.

## 4. Discussion

### 4.1. Summary of results

Our work compared the performance of four out-of-the-box NLP tools, one of which had two output configurations, across two different cohorts of data, focused on three phenotypes, namely, ischaemic stroke, SVD, and atrophy. Comparing the performance of the tool in two cohorts usually resulted in lower performance levels in the GS dataset. However, error analysis on false positives and negatives, looking across age groups and NHS health boards, revealed important insights into the tools and highlighted that the comparison of F1 scores should always be interpreted within the context of the different NLP tools and how they were developed. We found that three main factors affected tool performance, namely, uncertainty in language, particularly occurring in lower age groups; adaptability to different health board data; and training of the tools, including variations in annotation schema. Our work also showed the value of using image reads and labelled reports when comparing differing tools.

### 4.2. Language indicating diagnostic uncertainty

The GS cohort had a broader age range than NHS Fife, which was an elderly cohort, and we found that scan reports of younger people frequently used uncertain language, particularly for ischaemic stroke, e.g., “this may possibly be an ischemic infarct.” The use of uncertain language led to discrepancies between ground truth and NLP tool predictions and is a known problem for NLP using radiology reports, with studies such as Callen et al. (20) characterising where uncertain language is used. To address this issue, more consideration is needed when handling uncertainty in training NLP tools. In developing CheXpert data (chest radiology reports), Irvin et al. (21) investigated different approaches to incorporating uncertainty within model predictions. They showed that the approach, similar to ALARM+U, of treating uncertain outcomes as certain provides better prediction in detecting some phenotypes (e.g., atelectasis and oedema). However, for other phenotypes (e.g., cardiomegaly), using a model specifically trained to include uncertain labels allows for better disambiguation. It is also worth noting that adding uncertainty to ALARM+ for atrophy did not result in any difference in the prediction outcomes of ALARM+. A better understanding of how NLP tools can best approach uncertain language and studying how uncertain language is applied when describing specific phenotypes would help improve prediction models.

Uncertain language may also affect annotator labelling, as does the number of annotators used. The number of annotators can lead to inconsistency between annotators and amplify this issue when making decisions around uncertain language. Despite the use of guidelines for annotation, there is still subjectivity when it comes to deciding a label based on uncertain language (e.g., maybe, probably, or possibly). Work in the annotation field has suggested using annotator inconsistencies to improve annotation labelling (22). Chapman et al. (23), who developed ConTEXT, which looks for contextual features (negation, temporality, or who has experienced the condition, e.g., patient, family, and member), have shown how this may assist annotators when labelling by identifying these uncertain conditions to support classification. Other work, such as that by the ALARM+ authors, considered how a template method could improve understanding of uncertain terminology (18). They defined terminology that should be used to map to uncertain, positive, and negative entities, and this vocabulary was gathered throughout the annotation. The authors then used a template mechanism to explicitly teach the model how to change its prediction based on small changes in the language and phrasing. Given the subjectivity of radiology language, improving consistency and reliability of radiology report-labelling is important for development.

### 4.3. Different health board data

We observed that, in general, NLP tool performance across different NHS health board data impacted the rule-based system EdIE-R more, although this varied across phenotypes, with less impact seen on SVD and atrophy. This suggests that a neural-based model may be more generalisable for some phenotypes. There is potential for exploration with other types of machine learning models that may perform better across different data sources. Schrempf et al. (18) compared the EdIE-R and ALARM+ approaches on their dataset and found similar findings. However, the reason for the differences could relate to other variances in the training data, such as underlying population characteristics.

### 4.4. Annotation schema and training of tools

The datasets NLP tools are trained on and any annotation schema used for testing and refining the tool also influence tool performance. A poorer performance level may relate to differences in training data that the tools were exposed to. For example, being trained on data from different age groups affected the performance on SVD and atrophy phenotypes, which tend to be described in the report text in relation to whether they are “normal for age” or “advanced for age.” The rules of EdIE-R were designed based on the guidelines used by the annotators in this study. Therefore, EdIE-R outcomes will more readily align to the annotated labels than the other tools, e.g., Sem-EHR and ALARM+ predicted these as positive for atrophy and SVD. EdIE-R, Sem-EHR, and ALARM+ were trained on elderly cohorts (aged over 71 years), and this signifies the need to broaden the training exposure when developing a tool. We also observed that most ALARM+ false positives in ischaemic stroke and false negatives in SVD result from the annotation schema ALARM+ was trained on, which considered all mentions of atrophy to be positive including “normal for age,” compared with the annotation schema applied in our study. These differences in training and annotation make it difficult to adequately cross-compare tools. Moreover, these differences also highlight how adaptation or re-training would be needed to apply tools for purposes different from what they were originally designed for.

Clinical practice and guidelines will also impact how annotation is performed and phenotypes defined. For example, annotation protocols for the NLP tools may be specifically designed to identify phenotypes relevant to specific clinical scenarios (e.g., to fit with particular treatment guidelines or to identify people for clinical trials). In addition, clinical practice and the use of clinical guidelines may vary between settings, e.g., NHS health boards, and could determine what information is included in radiology reports and how they are presented. The clinical indication for imaging is another factor to consider. The GS dataset contains scans conducted for any indication. In contrast, the NHS Fife dataset contained scans conducted for people with delirium. Therefore, the reports may differ in content and focus. Variation in terminology and formatting is a recognised issue for NLP of radiology reports (Pons et al. 2016), and considering these contextual differences is important when comparing different bodies of radiological text.

### 4.5. Alternative methods for measuring performance

Interestingly, our comparison of the NLP tools to the research image reads removes much of the ambiguity around the tools trained on different annotation schemas. Accordingly, this showed that the results of the tools were much more similar, with relatively no difference between ALARM+ and EdIE-R for some phenotypes (e.g., atrophy). This demonstrated the value of using an alternative method to report-labelling when comparing tools developed on different annotation schemas. However, comparing the research image reads to the report annotated labels highlighted that this type of comparison is not without difficulties as more phenotypes are observed in the image than are written up in a single report. This is because a person may have multiple scans and a radiologist may only write up what has not been previously mentioned or may focus on a specific reason for the scan. Wood et al. (24) also reported similar differences between medical images and corresponding radiology reports.

### 4.6. Implication for clinical practice and NLP research

This work implies that if these tools are applied to clinical settings, it cannot be performed “out of the box.” In addition, it is essential to understand the context of their development to assess whether they are suitable for the task at hand or whether further training is required. For NLP practice, we highlighted several areas for future work to improve NLP tool performance. First, it is crucial to investigate uncertainty in language and understand how to deal with this for different phenotypes. Second, there is a need to understand annotation schemas that NLP tools are built on, particularly when comparing tools, and investigate alternative approaches, such as brain image reads, which could support evaluation and promote confidence in using NLP tools.

Works such as Mitchell et al. (9) and Bender and Friedman (10) promote frameworks for understanding cases of model use, outlining the contexts where a model is suited to a specific use and ensuring transparency about data characteristics used when models are developed. Application of such frameworks in health-related NLP tools is scarce. However, if published alongside models, they could support stronger claims of how these models would generalise to unseen data and how the results can be reproduced. For example, it would have been useful to have a more detailed understanding of the characteristics of the data all four models were trained on, as the level of detail differed within each published paper and was not comparable, e.g., age bands of patients. Regularly adopting these frameworks would also support the deployment and use of NLP tools in clinical use. Model developers could use the framework to more explicitly define the parameters within which a model was developed and expected to operate within to maintain the performance level.

Finally, there is a continuing need to pursue access to radiology reporting datasets using additional demographic variables to test and refine these tools on alternative, unseen data.

### 4.7. Limitations

The comparison of the models in this study has limitations because of the restricted access to data in our cohorts. We did not have access to ethnicity or specific geographical details besides the NHS health board. In addition, because of the restrictions imposed by the bodies that release health data, we have no way of establishing if some of the data used in this study from either cohort could have been within the data used when developing tools. In addition, we specified that this was a document-level study at the outset. Although ALARM+ predicts at the sentence level, we point out that it would have been time-consuming and out of the scope of the paper to do a sentence-level comparison to annotation labels. Further analysis of the NHS Fife data is no longer possible because of the access restrictions and additional costs. It is also impossible to do sentence-level comparisons against the image reads as image reads provide a complete output of the entire image and can only be compared at the document output level.

## 5. Conclusions

We conclude that the different NLP tools vary in F1 (and precision/recall) scores for the three phenotypes, especially for ischaemic stroke compared with SVD or atrophy. Several factors influence the performance of the NLP tools: uncertainty in language, which occurs more for younger patient age groups; the data and annotations that a model was trained on; and the different NHS trusts from which data was obtained. If these tools are to be applied to clinical settings, it is important to understand the context of their development to assess if they are suitable for the task and environment they are being applied within.

## Data Availability

The datasets for this article are not publicly available due to concerns regarding patient anonymity. Requests to access the Generation Scotland dataset can be made via the following website: https://www.ed.ac.uk/generation-scotland/for-researchers/access.

## Footnotes

^1^ https://en.wikipedia.org/wiki/NHS_Scotland

^2^ https://www.ed.ac.uk/generation-scotland

^3^ http://www.nltk.org

^4^ https://www.ltg.ed.ac.uk/software/EdIE-R/

^5^ https://github.com/CogStack/CogStack-SemEHR

^6^ https://icaird.com/about/

^7^ https://docs.scipy.org/doc/scipy/reference/stats.html
