# Supplementary material for: Understanding the performance and reliability of NLP tools: a comparison of four NLP tools predicting stroke phenotypes in radiology reports
Source: Front Digit Health. 2023 Sep 28;5:1184919. doi: 10.3389/fdgth.2023.1184919 (PMC10569314; doi:10.3389/fdgth.2023.1184919)
Supplement: Supplementary file 1 [file Datasheet1.docx]

# Supplementary Material File 1

| Annotation Labels | **NHS Tayside** **& Fife** | **NHS Lothian** | **NHS Glasgow Greater** **Clyde** | | **NHS Grampian** | |
| --- | --- | --- | --- | --- | --- | --- |
| Annotator No.s | **1&4** | **1&2** | **1&2** | **1&3** | **1&2** | **1&3** |
| **Ischaemic Stroke** |  |  |  |  |  |  |
| *Cortical old* | 0 | 0.9 | 1 | 0.66 | - | 1 |
| *Cortical recent* | - | 1 | - | 1 | 1 | 1 |
| *Deep old* | 0.66 | 0.92 | 0.85 | 1 | 1 | 0.5 |
| *Deep recent* | - | 0.80 | - | 1 | 1 | - |
| *Underspecified* | 1 | - | - | 1 | 1 | 1 |
| **Small Vessel Disease** | 0.85 | 0.93 | 0.9 | 0.92 | 1 | 1 |
| **Atrophy** | 1 | 0.85 | 0.96 | 1 | 1 | 1 |

Table 1 Supplemental 1 Kappa for inter-annotator agreement for each NHS healthboard in Generation Scotland

| **Annotation Labels** | **NHS Fife** |
| --- | --- |
| **Ischaemic Stroke** |  |
| *Cortical old* | 0.84 |
| *Cortical recent* | 1 |
| *Deep old* | 0.97 |
| *Deep recent* | 0.0 (only 3) |
| *Underspecified* | 0.0  (only1) |
| **Small Vessel Disease** | 0.95 |
| **Atrophy** | 1 |

Table 2 Supplemental 1, Kappa values for inter-annotator agreement NHS Fife
