# Supplementary material for: Understanding the performance and reliability of NLP tools: a comparison of four NLP tools predicting stroke phenotypes in radiology reports
Source: Front Digit Health. 2023 Sep 28;5:1184919. doi: 10.3389/fdgth.2023.1184919 (PMC10569314; doi:10.3389/fdgth.2023.1184919)
Supplement: Supplementary file 3 [file Datasheet3.docx]

# Supplemental 3

| Cohorts |  | EdIE-R | Sem-EHR | ESPRESSO | ALARM+ | ALARM+U |
| --- | --- | --- | --- | --- | --- | --- |
| NHSFife | F1 | **0.98** | 0.74 | 0.74 | 0.90 | 0.90 |
|  | P | 0.97  (0.94-0.98) | 0.61  (0.56 -0.65) | **0.98**  (0.95 -0.99) | 0.86  (0.82 -0.90) | 0.82  (0.78 -0.86) |
|  | R | **0.98**  (0.96-0.99) | 0.96  (0.93 -0.98) | 0.59  (0.53 -0.65) | 0.94  (0.90-0.96) | **0.98**  (0.96 -0.99) |
| Generation Scotland | F1 | **0.93** | 0.66 | 0.77 | 0.87 | 0.83 |
|  | P | 0.88  (0.84 -0.92) | 0.50  (0.45-0.54) | **0.90**  (0.85 -0.93) | 0.80  (0.75 – 0.84) | 0.71  (0.66 -0.75) |
|  | R | 0.98  (0.94-0.98) | 0.97  (0.94 -0.99) | 0.68  (0.62 – 0.73) | 0.97   (0.94 -0.98) | **0.99**  (0.97 -1.0) |

Table 1 supplemental 3 Performance F1, precision, recall and 95% CI for Ischaemic Stroke for all NLP tools and both cohorts

| Age Group |  | EdIE-R | Sem-EHR | ESPRESSO | ALARM+ | ALARM+U |
| --- | --- | --- | --- | --- | --- | --- |
| NHSFife | F1 | **0.98** | 0.92 | 0.77 | 0.91 | 0.91 |
|  | P | **0.99**  (0.98 -1.0) | 0.92  (0.89 -0.94) | **0.99**  (0.95 -0.99) | **0.99**  (0.98 -1.0) | **0.99**  (0.98 -1.0) |
|  | R | **0.97**  (0.94-0.98) | 0.91  (0.88 -0.93) | 0.64  (0.53 -0.65) | 0.84  (0.80 -0.87) | 0.85  (0.82 -0.88) |
| Generation Scotland | F1 | **0.98** | 0.81 | 0.78 | 0.90 | 0.90 |
|  | P | **0.98**  (0.96-0.99) | 0.72  (0.68-0.76) | 0.90  (0.86 -0.93) | 0.96   (0.93 -0.97) | 0.96   (0.93 -0.97) |
|  | R | **0.97**  (0.95-0.99) | 0.92  (0.89 -0.94) | 0.68  (0.63 -0.73) | 0.84  (0.80 -0.88) | 0.86  (0.82 -0.89) |

Table 2 Supplemental 3 Performance F1, precision, recall and 95% CI for small vessel disease for all NLP tools and both cohorts

| Cohort |  | **EdIE-R** | **Sem-EHR** | **ALARM+** | **ALARM+U** |
| --- | --- | --- | --- | --- | --- |
| NHSFife | F1 | **0.99** | 0.89 | **0.99** | **0.99** |
|  | P | **1.0**  (0.98-1.0) | 0.93  (0.90-0.95) | 0.99  (0.97 -0.99) | 0.99  (0.97 0.99) |
|  | R | 0.99  (0.98-1.0) | 0.85  (0.81 -0.88) | **1.0**  (0.99 -1.0) | **1.0**  (0.99 -1.0) |
| Generation Scotland | F1 | **0.96** | 0.73 | 0.91 | 0.91 |
|  | P | **0.99**  (0.97 -1.0) | 0.66  (0.62-0.71) | 0.84  (0.80 – 0.87) | 0.84  (0.80 -0.87) |
|  | R | 0.93  (0.90-0.96) | 0.82  (0.77 -0.86) | **1.0**  (0.98 -1.0) | **1.0**   (0.98 -1.0) |

Table 3 Supplemental 3 Performance F1, precision, recall and 95% confidence intervals for all NLP tools across both cohorts for atrophy

| Age Group |  | EdIE-R | Sem-EHR | ESPRESSO | ALARM+ | ALARM+U |
| --- | --- | --- | --- | --- | --- | --- |
| <50 | F1 | 0.89 | 0.57 | 0.63 | **0.92** | 0.80 |
|  | P | 0.80  (0.54-0.93) | 0.43  (0.30-0.63) | **0.86**  (0.47-0.97) | **0.86**  (0.60-0.96) | 0.67  (0.43-0.84) |
|  | R | **1.0**  (0.75-1.0) | 0.83  (0.55-0.95) | 0.5  (0.25-0.75) | **1.0**  (0.75-1.0) | **1.0**  (0.75-1.0) |
| 50-70 | F1 | **0.92** | 0.61 | 0.73 | 0.87 | 0.81 |
|  | P | 0.88  (0.79-0.93) | 0.44  (0.37-0.51) | **0.91**  (0.81-0.96) | 0.80  (0.72-0.87) | 0.69  (0.60-0.76) |
|  | R | 0.97  (0.90-0.99) | **1.0**  (0.96-1.0) | 0.61  (0.50-0.71) | 0.94  (0.87-0.97) | 0.99  (0.94-1.0) |
| 71+ | F1 | **0.91** | 0.70 | 0.81 | 0.90 | 0.84 |
|  | P | 0.84  (0.74-0.91) | 0.54  (0.48-0.60) | **0.89**  (0.82-0.94) | 0.79  (0.72-0.84) | 0.72  (0.66-0.78) |
|  | R | 0.98  (0.92-1.0) | 0.97  (0.92-0.98) | 0.73  (0.66-0.80) | 0.98  (0.94-0.99) | **0.99**  (0.96-1.0) |

Table 4 Supplemental 3 F1, precision, recall and 95%confidence intervals for each age group in the Generation Scotland data across all NLP tools, ischaemic stroke

| Health Board |  | EdIE-R | Sem-EHR | ESPRESSO | ALARM+ | ALARM+U |
| --- | --- | --- | --- | --- | --- | --- |
| Tayside &Fife | F1 | **1.0** | 0.77 | 0.76 | 0.86 | 0.85 |
|  | P | **1.0**  (0.94-1.0) | 0.63  (0.52-0.72) | 0.97  (0.85-0.99) | 0.79  (0.67-0.87) | 0.73  (0.62-0.82) |
|  | R | **1.0**  (0.94-1.0) | **1.0**  (0.93-1.0) | 0.61  (0.49-0.74) | 0.95  (0.85-0.98) | **1.0**  (0.94-1.0) |
| Lothian | F1 | **1.0** | 0.42 | 0.73 | 0.87 | 0.81 |
|  | P | **1.0**  (0.76-1.0) | 0.32  (0.17-0.52) | 0.89  (0.55-0.97) | 0.76  (0.52-0.90) | 0.68  (0.46-0.85) |
|  | R | **1.0**  (0.77-1.0) | 0.61  (0.35-0.82) | 0.62  (0.35-0.82) | **1.0**  (0.77-1.0) | **1.0**  (0.77-1.0) |
| GGC | F1 | **0.89** | 0.67 | 0.75 | 0.88 | 0.92 |
|  | P | 0.82  (0.74-0.88) | 0.50  (0.42-0.56) | **0.84**  (0.75-0.90) | 0.81  (0.73-0.87) | 0.69  (0.61-0.76) |
|  | R | 0.98  (0.93-0.99) | **0.99**  (0.95-1.0) | 0.68  (0.58-0.76) | 0.97  (0.91-0.98) | **0.99**  (0.95-1.0) |
| Grampian | F1 | **0.91** | 0.63 | 0.82 | 0.87 | 0.83 |
|  | P | 0.88  (0.79-0.93) | 0.46  (0.39-0.54) | **0.93**  (0.84-0.97) | 0.79  (0.69-0.86) | 0.71  (0.62-0.79) |
|  | R | 0.95  (0.87-0.98) | **0.99**  (0.93-1.0) | 0.74  (0.63-0.82) | 0.97  (0.91-0.99) | **0.99**  (0.93-1.0) |

Table 5 Supplemental 3 F1, precision, recall and 95% confidence intervals for performance on each NHS healthboard in Generation Scotland cohort, ischaemic stroke

| Age Group |  | EdIE-R | Sem-EHR | ESPRESSO | ALARM+ | ALARM+U |
| --- | --- | --- | --- | --- | --- | --- |
| <50 | F1 | **0.89** | 0.08 | 0.29 | 0.4 | 0.4 |
|  | P | 0.80  (0.36 -0.97) | 0.05  (0.01-0.24) | 0.34  (0.07-0.81) | **1.0**  (0.16-0.99) | **1.0**  (0.16-0.99) |
|  | R | **1.0**  (0.48 -0.99) | 0.25  (0.05-0.77) | 0.30  (0.05-0.71) | 0.25  (0.05-0.71) | 0.25  (0.05-0.72) |
| 50-70 | F1 | **0.96** | 0.72 | 0.73 | 0.85 | 0.86 |
|  | P | 0.95  (0.90-0.98) | 0.59  (0.52-0.66) | 0.85  (0.76-0.91) | **0.96**  (0.90-0.99) | 0.95  (0.89-0.98) |
|  | R | **0.96**  (0.91-0.98) | 0.93  (0.87-0.97) | 0.64  (0.60-0.73) | 0.76  (0.67-0.83) | 0.79  (0.70-0.85) |
| 71+ | F1 | **0.98** | 0.88 | 0.80 | 0.92 | 0.93 |
|  | P | **0.98**  (0.95-1.0) | 0.85  (0.80-0.88) | 0.93  (0.89-0.96) | 0.96  (0.92-0.97) | 0.96  (0.92-0.98) |
|  | R | **0.98**  (0.94-1.0) | 0.93  (0.88-0.95) | 0.70  (0.65-0.75) | 0.88  (0.84-0.92) | 0.90  (0.85-0.93) |

Table 6 Supplemental 3 F1, precision and recall including 95% confidence intervals for all tools across the age groups in Generation Scotland, Small vessel disease

| Health Board |  | EdIE-R | Sem-EHR | ESPRESSO | ALARM+ | ALARM+U |
| --- | --- | --- | --- | --- | --- | --- |
| Tayside &Fife | F1 | **0.99** | 0.73 | 0.68 | 0.83 | 0.83 |
|  | P | 0.98  (0.89-0.99) | 0.65  (0.52-0.75) | 0.77  (0.85-0.99) | **1.0**  (0.89-1.0) | **1.0**  (0.89-1.0) |
|  | R | **1.0**  (0.92-1.0) | 0.84  (0.81-0.92) | 0.6  (0.49-0.74) | 0.71  (0.57-0.82) | 0.71  (0.57-0.82) |
| Lothian | F1 | **1.0** | 0.49 | 0.88 | 0.88 | 0.91 |
|  | P | **1.0**  (0.86-1.0) | 0.48  (0.30-0.67) | **1.0**  (0.83-1.0) | **1.0**  (0.83-1.0) | **1.0**  (0.84-1.0) |
|  | R | **1.0**  (0.86-1.0) | 0.5  (0.31-0.69) | 0.79  (0.59-0.90) | 0.79  (0.59-0.91) | 0.83  (0.64-0.93) |
| GGC | F1 | **0.97** | 0.88 | 0.67 | 0.91 | 0.92 |
|  | P | **0.96**  (0.92-0.98) | 0.81  (0.75-0.86) | 0.87  (0.79-0.92) | 0.94  (0.89-0.97) | 0.93  (0.89-0.96) |
|  | R | **0.97**  (0.93-0.99) | **0.97**  (0.93-0.99) | 0.57  (0.49-0.64) | 0.89  (0.83-0.93) | 0.91  (0.85-0.94) |
| Grampian | F1 | **0.98** | 0.80 | 0.89 | 0.89 | 0.90 |
|  | P | **1.0**  (0.97-1.0) | 0.69  (0.62-0.75) | 0.96  (0.91-0.98) | 0.97  (0.92-0.99) | 0.97  (0.92-0.99) |
|  | R | **0.96**  (0.92-0.98) | 0.95  (0.90-0.97) | 0.83  (0.76-0.89) | 0.83  (0.76-0.89) | 0.85  (0.77-0.90) |

Table 7 Supplemental 3 F1, Precision and recall including 95% confidence intervals for all tools across NHS health boards in Generation Scotland, small vessel disease

| Age Group |  | EdIE-R | Sem-EHR | ALARM+ | ALARM+U |
| --- | --- | --- | --- | --- | --- |
| <50 | F1 | **0.94** | 0.26 | 0.72 | 0.72 |
|  | P | **0.89**  (0.55-0.97) | 0.16  (0.07-0.33) | 0.57  (0.32-0.78) | 0.57  (0.32-0.79) |
|  | R | **1.0**  (0.66-1.0) | 0.63  (0.30-0.86) | **1.0**  (0.66-1.0) | **1.0**  (0.66-1.0) |
| 50-70 | F1 | **1.0** | 0.68 | 0.87 | 0.87 |
|  | P | **0.99**  (0.94-1.0) | 0.60  (0.48-0.65) | 0.78  (0.70-0.85) | 0.78  (0.70-0.85) |
|  | R | 0.96  (0.89-0.98) | 0.84  (0.75-0.90) | **0.99**  (0.94-1.0) | **0.99**  (0.94-1.0) |
| 71+ | F1 | **0.96** | 0.80 | 0.93 | 0.93 |
|  | P | **0.99**  (0.95-1.0) | 0.78  (0.72-0.83) | 0.88  (0.83-0.91) | 0.88  (0.82-0.91) |
|  | R | 0.92  (0.86-0.96) | 0.82  (0.76-0.86) | **1.0**  (0.98-1.0) | **1.0**  (0.98-1.0) |

Table 8 Supplemental 3 F1, precision, recall and 95% confidence intervals for age groups across the Generation Scotland cohort for all NLP tools, atrophy

| Health Board |  | EdIE-R | Sem-EHR | ALARM+ | ALARM+U |
| --- | --- | --- | --- | --- | --- |
| Tayside &Fife | F1 | **0.98** | 0.79 | 0.96 | 0.96 |
|  | P | **1.0**  (0.94-1.0) | 0.77  (0.66-0.85) | 0.93  (0.85-0.97) | 0.93  (0.85-0.97) |
|  | R | 0.95  (0.87-0.98) | 0.82  (0.71-0.89) | **1.0**  (0.95-1.0) | **1.0**  (0.95-1.0) |
| Lothian | F1 | **1.0** | 0.29 | 0.81 | 0.81 |
|  | P | **1.0**  (0.77-1.0) | 0.23  (0.10-0.44) | 0.68  (0.46-0.85) | 0.68  (0.46-0.85) |
|  | R | **1.0**  (0.77-1.0) | 0.38  (0.18-0.65) | **1.0**  (0.77-1.0) | **1.0**  (0.77-1.0) |
| GGC | F1 | **0.95** | 0.70 | 0.93 | 0.93 |
|  | P | **0.98**  (0.92-0.98) | 0.63  (0.56-0.69) | 0.87  (0.81-0.91) | 0.87  (0.81-0.91) |
|  | R | 0.92  (0.93-0.99) | 0.77  (0.70-0.83) | **0.99**  (0.97-1.0) | **0.99**  (0.97-1.0) |
| Grampian | F1 | **0.97** | 0.83 | 0.87 | 0.87 |
|  | P | **0.93**  (0.96-1.0) | 0.74  (0.65-0.81) | 0.77  (0.69-0.84) | 0.77  (0.69-0.84) |
|  | R | 0.98 | 0.89 | **1.0**  (0.96-1.0) | **1.0**  (0.96-1.0) |

Table 9 Supplemental 3 F1, precision, recall and 95% confidence intervals for atrophy for all tools across Generation Scotland NHS health boards, atrophy
